# Intelligent Semantic Segmentation for Self-Driving Vehicles Using Deep Learning

**DOI:** 10.1155/2022/6390260

**Published:** 2022-01-17

**Authors:** Qusay Sellat, SukantKishoro Bisoy, Rojalina Priyadarshini, Ankit Vidyarthi, Sandeep Kautish, Rabindra K. Barik

**Affiliations:** ^1^Department of Computer Science and Engineering, C.V. Raman Global University, Bhubaneswar, India; ^2^Department of CSE&IT, Jaypee Institute of Information Technology, Noida, India; ^3^LBEF Campus Kathmandu, Kathmandu, Nepal; ^4^School of Computer Applications, KIIT Deemed to be University, Bhubaneswar, India

## Abstract

Understanding the situation is a critical component of any self-driving system. Accurate real-time visual signal processing to create pixelwise classed pictures, also known as semantic segmentation, is critical for scenario comprehension and subsequent acceptance of this new technology. Due to the intricate interaction between pixels in each frame of the received camera data, such efficiency in terms of processing time and accuracy could not be achieved prior to recent advances in deep learning algorithms. We present an effective approach for semantic segmentation for self-driving automobiles in this study. We combine deep learning architectures like convolutional neural networks and autoencoders, as well as cutting-edge approaches like feature pyramid networks and bottleneck residual blocks, to develop our model. The CamVid dataset, which has undergone considerable data augmentation, is utilised to train and test our model. To validate the suggested model, we compare the acquired findings to various baseline models reported in the literature.

## 1. Introduction

Being able to move efficiently and safely in vehicles that are driverless has been a hot research topic in recent years, and many companies and research centres are trying to come up with the first completely practical driverless car model. This is a very promising field with a lot of possible benefits such as increase of safety, less costs, comfortable travel, increased mobility, and reduced environmental footprint [1]. Semantic segmentation is the process of assigning each pixel of the received image into one of the predefined classes. These classes represent the segment labels of the image, e.g., roads, cars, signs, traffic lights, or pedestrians [2]. Therefore, semantic segmentation is sometimes referred to as “pixelwise classification.” The main benefit of semantic segmentation is situation understanding. It is therefore used in many fields such as autonomous driving, robotics, medical images, satellite images, precision agriculture, and facial images as a first step to achieving visual perception. Autonomous driving depends on the information received by sensors of the surrounding environment in order to form a complete picture of the driving situation. Because the visual signal is very rich in such information, doing semantic segmentation correctly is crucial for scene understanding. The more we perform semantic segmentation with a high accuracy and a short time, the more correctly the ego vehicle understands the surrounding environment and accordingly make the right decision every moment. However, semantic segmentation is challenging due to the complicated relationship between pixels in each image frame and also between successive frames. Even with the fast development of new technologies such as deep learning which have made the mission of semantic segmentation more efficient, doing accurate semantic segmentation in real time is still a hot topic in current research as shown in detail later.

In this paper, we benefit from deep learning methods, especially convolutional neural networks (CNNs) and autoencoders (AEs) in order to design an accurate, real-time semantic segmentation model. The main contributions of this paper are as follows:

- Designing an accurate and real-time semantic segmentation system for self-driving cars by taking advantage of two main deep learning architectures: CNNs and AEs. A hybrid model based on the concepts of feature pyramid networks (FPNs) and bottleneck residual blocks is built to perform the semantic segmentation efficiently.
- The proposed model is trained and tested using CamVid common dataset for semantic segmentation for self-driving missions. Extensive data augmentation has been done to the training set in order to overcome the problem of the small size of semantic segmentation datasets for autonomous driving.

## 2. Related Work

Thanks to the rapid improvements in deep learning research last decade, great results have been achieved in the field of computer vision. Developing CNNs [3] had the biggest impact on this success as tasks such as object recognition and detection have witnessed a huge jump in accuracy and speed. After the success of the early CNN models such as LeNet [4] and AlexNet [5] (AlexNet was proposed in 2012 but published in 2017), the number of proposed CNN works exploded. VGG [6], with its large number of parameters, performed well on the ImageNet dataset [7]. It increased the used number of hidden layers to 16 or 19 weight layers. At the same time, Inception [8] used the principle of network-in-network [9] to increase the depth of the CNN to 22 trainable layers. As the depth of the neural network increased, serious problems such as gradient vanishing and gradient exploding surfaced. Later proposals tried to overcome these problems by developing new techniques such as skip connections that were designed in the shape of additional connections like in ResNets [10] or the shape of concatenation connections like in DenseNets [11]. However, in addition to the gradient problems, increasing the number of layers and trainable parameters made the use of these models limited, and any idea of implementing them within constrained environments or real-time systems is impractical. Xception [12], ShuffleNet [13], MobileNetV1 [14], and MobileNetV2 provided a convenient way of designing real-time CNNs by focusing on the use of depthwise separable convolutions [15]. This allowed researchers to design mobile vision applications that are both accurate and real time. Other solutions such as EfficientNets [16] made it possible to compromise between the performance of the designed model and its complexity.

Similar to other computer vision topics, semantic segmentation research has experienced a huge improvement in the era of deep learning. In addition to CNNs, AEs were used to design semantic segmentation models that are much more efficient than old models. Recent semantic segmentation research focused on convolutional autoencoders (CAEs) which are autoencoders whose encoder and decoder parts are convolutional and deconvolutional layers, respectively. CNN models that were developed initially for object recognition and detection have been used as the backbone architectures of CAEs developed for semantic segmentation. FCN [17] used fully convolutional architecture with a large number of parameters to perform semantic segmentation. It was one of the first attempts towards getting rid of fully connected layers. SegNet [18] and SegNet-Basic [19] used VGG architecture as a backbone for the encoder and the decoder. It used the pooling indices of the encoder for the upsampling operation in the decoder. Some other architectures such as UNet [20] used some kind of skip connections between the encoder and the decoder and some other techniques such as data augmentation to increase segmentation accuracy.

Although accuracy of semantic segmentation models improved thanks to the above-mentioned models and some other architectures such as PSPNet [21], Dilated [22], and DeepLab [23], real-time semantic segmentation is still a hot research area, especially that some fields such as autonomous driving and robotics require very accurate semantic segmentation with a minimum amount of processing time. Because images are rich in semantic information, a significant number of trainable parameters are required to capture the complexity of possible images and it is very hard to develop lite segmentation models without sacrificing accuracy.

Some models such as FPN were designed with a smaller number of parameters. However, despite being efficient in the semantic segmentation missions, the encoder architecture used in the original FPN model has a structure similar to ResNets which can cause problems when generalized to work in real-time conditions. Super-lighter models such as ApesNet [24], Enet [25], ESPNet [26], and ESCNet [27] tried to minimize the number of parameters so that the semantic segmentation can be done in real time or embedded systems. Despite the fact that these models provided practical solutions to satisfy the real-time condition, crucial applications such as road scene understanding in autonomous vehicles need much more segmentation accuracy.

## 3. Preliminaries and Discussion

### 3.1. Feature Pyramid Network

The term “feature pyramid” is used in computer vision tasks to describe the process of extracting features from images in a hierarchical manner, i.e., high-level features are extracted along with low-level ones. Using this approach, a model is able to recognize objects correctly even if these objects appear in various scales. The main idea of the feature pyramid can be used to develop an end-to-end approach for semantic segmentation so that the designed algorithm produces semantically strong levels for each image. For this reason, in an end-to-end model such as ConvNets, a feature hierarchy is built layer by layer. Multiscale spatial resolutions can be built this way [28].

**Figure 1:**
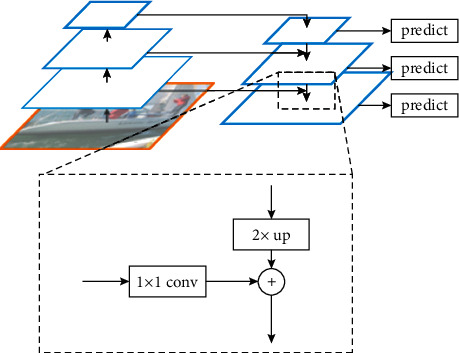
Architecture of feature pyramid network.

A feature pyramid network is a CAE that consists of two parts: bottom-up module (encoder) and top-down module (decoder). In the bottom-up module, the forward propagation of the used backbone architecture is performed. Using this backbone, a feature hierarchy is obtained. This hierarchy comprises the feature maps of several scales. A group of layers that produces output maps of the same size is said to form a “stage” so that each stage represents one pyramid level. Feature hierarchy ideas can be invested in the field of semantic segmentation by combining the low-level features with high-level features in a top-down module and skip connections. This module upsamples spatially coarser feature maps from higher pyramid levels and then tunes them with features from the bottom-up module [29, 30]. Each skip connection merges between two feature maps of the same dimensions so that one comes from the bottom-up path and the other one comes from the top-down path. Scaling ratio of 2 is used in the original FPN architecture and also in ours. So, in the bottom-up module, some stages are downsampled by a factor of 2 compared to the previous one. Also, in the top-down module, the respective stages are upsampled by a factor of 2 compared to the previous one.  Figure 1 shows the architecture of feature pyramid network consisting of bottom-up and top-down modules representing feature hierarchy consisting of feature maps [31]. In addition, in some stages of the top-down path, a (1 ∗ 1) convolution is used in the skip connection to change the channel dimension of the used feature map before merging it with the coming upsampled map of the previous top-down stage in an elementwise addition manner. The channel dimension of the top-down module is referred to as a matrix of dimension *d*.

### 3.2. Bottleneck Residual Network

Traditional convolutional blocks have a high computational complexity that makes them hard to apply in real-time applications. One approach suggests replacing them with depthwise separable convolutions [15] that approximately have the same performance but much less complex than traditional ones. A depthwise separable convolution block is built by splitting the normal convolutional layer into two modules:

- A depthwise convolution layer that processes the inputs by filtering them through a 3 ∗ 3 convolution. This layer applies a single filter for each input channel. The re-scaling of spatial dimensions may be made in this module.
- A pointwise convolution layer that combines these filtered values to create new features through a 1 ∗ 1 convolution. This layer combines the outputs of the depthwise convolution layer. The re-scaling of the channel dimension may be made in this module [31].

In addition, when depthwise separable convolution blocks are used to build deep neural networks, no pooling layers are used. Strides are used alternatively for downsampling tasks. Depthwise separable convolutions are used in MobileNetV1 [14]. 13 blocks of this type are used in MobileNetV1 initial configuration.

A bottleneck residual block is a slightly modified version of depthwise separable convolution blocks. A third module that is called an “expansion layer” is added. The expansion layer increases the number of channels of the input that come from the previous bottleneck block. The default expansion factor is 6. In addition, instead of a pointwise convolution layer, a bottleneck residual block has a “projection layer” that reduces the number of channels (compared to pointwise convolution layer that normally increases the number of channels). No significant change was applied to the design of the depthwise convolution layer [32–34].

In both depthwise separable convolution and bottleneck residual block, a batch normalization layer is used after each convolution process. Also, ReLU6 is used as the activation function instead of the normal ReLU. RelU6 is used as the activation function of each layer excluding the projection layer, where designers found that using a nonlinear function after this layer can make the performance worse. Additionally, in a similar manner to ResNets [10], a skip connection that links between the input of the first layer of some bottleneck residual blocks and the output of the last layer of the same block is used to overcome the problem of gradient vanishing. This connection is called a “residual connection.”

Bottleneck residual blocks significantly reduce the number of computations that are needed for processing the input because of the projection layers. Because both expansion and projection layers contain learnable parameters, the useful information is transmitted to deeper layers with only little loss. Also, residual connections keep the learning process controlled and assure that useful information from the earlier layers are received by former layers. The main benefit of bottleneck residual blocks is that they reduce the amount of the data flow in the model. Because of having expansion and projection layers, the whole computation is done on an expanded (uncompressed) version of data, while the data flow between bottleneck residual blocks is minimized (a compressed version of data passes through the model).

**Figure 2:**
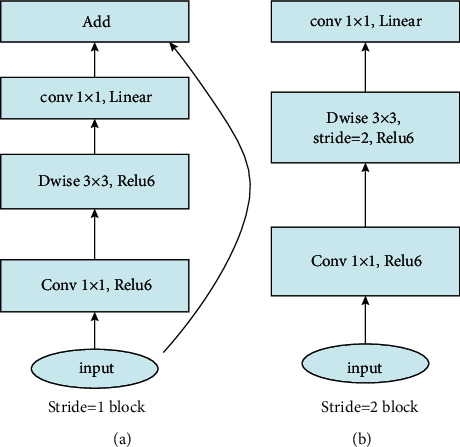
Types of bottleneck residual blocks (BRBs). (a) BRB does not perform spatially nor channel dimension reduction. (b) BRB performs both, and thus no residual connection is allowed.

It is worth noting that instead of using pooling layers to decrease the spatial dimensions of data as most architectures do, MobileNetV2 uses strides for spatial reduction. A stride value *s* = 2 is used in the depthwise convolution layer when this type of reduction is needed. MobileNetV2 also does the reduction of channel dimension in the same block by making the channel dimension of the output of the projection layer less than the channel dimension of its input. In this case, no residual connection is applied to the containing bottleneck residual block as the spatial and channel dimensions of the input of the block are different from the respective dimensions of the output of the block. In case spatial and channel dimensions are preserved, a stride value *s* = 1 is used in the depthwise convolution layer and residual connection is still applied. This small difference between the two types of blocks is further explained in Figure 2. Bottleneck residual blocks are the building blocks in MobileNetV2 [31] which by default uses 17 consecutive layers of this type in its architecture (in addition to the initial convolution layer of 32 filters).

Table 1 shows more detailed specifications where *h* and *w* are the input spatial dimensions, *k* and *k*′ are the numbers of input and output channels, respectively, *s* is the stride value, and *t* is the expansion factor.

## 4. Proposed Model Architecture

To come up with a practical semantic segmentation model for self-driving cars, it is crucial to consider two things. First, it has to be able to work in real-time conditions. Second, it has to be accurate enough so that the driverless car can depend on its results to understand the surrounding environment.

We design a model that uses the basic concepts of FPN and bottleneck residual network. The general architecture of the model is similar to FPN where we have a CAE that consists of a bottom-up path (encoder) and a top-down path (decoder) in line with the modern designs that comprise two parts: an encoder and a decoder.

However, despite being efficient in the semantic segmentation missions, the encoder architecture used in the original FPN model has a structure similar to ResNets [32] which can cause problems when generalized to work in real-time conditions.

To design an efficient model having similar working like traditional FPN model that can also perform well in real-time situations, we build the encoder part of the model so that it is similar to MobileNetV2 that is specifically designed for efficient real-time processing. As in MobileNetV2, the encoder part includes many bottleneck residual blocks (Figure 2) in a row. Here the residual connections resided in between the bottleneck layer. The input and output also consist of thin bottleneck layer. It makes use of light weight depthwise convolutional filters which help to reduce the computation time.

**Figure 3:**
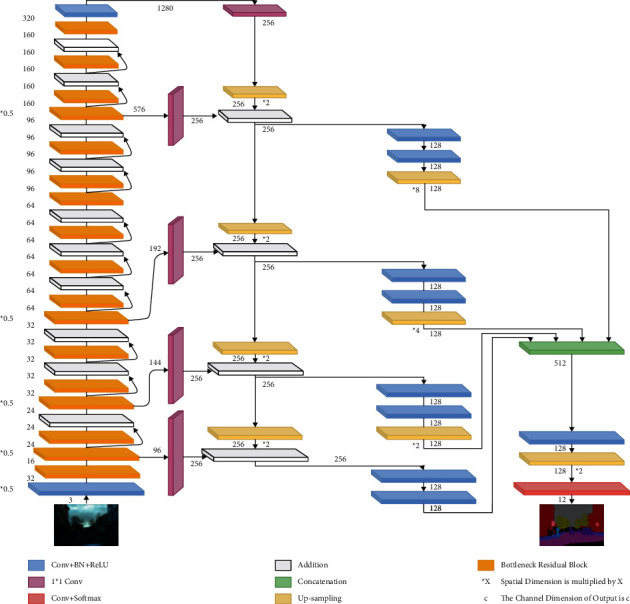
Proposed architecture of the bottleneck residual block.

A detailed illustration of the architecture of our hybrid model is shown in Figure 3, where the bottleneck residual blocks are present in the left. The number of dimensions decided as presented is inferred by convolutional operators. We notice the two main parts of the model. The first one is the bottom-up part. It comprises many stages. Each stage is formed of one or more bottleneck residual blocks (this is the main difference from the original FPN model where ResNet structures instead of MobileNetV2 are used inside these stages). Similarly, the top-down path comprises many stages. The number of channel dimensions in each stage in the top-down path is *d* = 256 before halving it to *d* = 128. All the updations to the number of dimensions are performed by using the trainable parameters using convolutional operations in deep model. Skip connections link some stages in the bottom-up path with the corresponding stage in the top-down path. Finally, the outputs of all top-down stages are concatenated before the prediction process which depends on a softmax layer to predict the class label of each pixel of the input image.

### 4.1. Dataset

For training and testing purposes, we use Cambridge-driving Labeled Video Database (CamVid) [20, 35] which is a very common dataset for research about vision in self-driving cars. CamVid was captured from the perspective of a driving automobile. It contains video sequences of various road scenes. For semantic segmentation research purposes, a subset of these scenes was labeled at the pixel level so that each pixel was assigned to some predefined class label.

**Figure 4:**
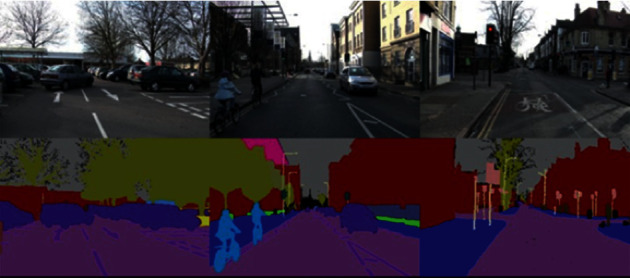
Some samples of the CamVid dataset [20].

The version of the CamVid dataset we use in this research is identical to that used in modern semantic segmentation research [2, 18]. It was extracted from 5 video sequences taken at 30 Hz, so that ground truth was provided at 1 Hz (i.e., they labeled 1 frame out of 30 frames in each second). The total number of images in this version is 701: 367 images in the training set, 101 images in the validation set, and 233 images in the testing set. Each image frame has a dimension of 360480. The group of semantic classes contains 11 different classes (in addition to the void class). These classes represent the main labels of semantic segments that every road scene can normally be divided into (sky, pole, building, road, tree, sidewalk, sign symbol, car, fence, bicyclist, and pedestrian). Some samples of the CamVid dataset are shown in Figure 4. For presentation purposes, different colors were assigned to the different class labels so that each semantic segment can be visually distinguished from other segments.

Because of the nature of the captured video sequences representing realistic road scenes, there is a significant amount of imbalance in class frequencies which makes training of deep neural architectures on CamVid road scenes a big challenge for researchers. For example, classes such as sky, road, and building are 40–50 times more frequent than other classes such as bicycles and sign symbols. However, using realistic datasets such as CamVid to train and test proposed models is inevitable if we want to make our models able to be integrated into real-world self-driving systems. Because of that, we choose CamVid in our research and try to overcome the problem of class imbalance by using a common practice in the training phase of deep neural networks which is class weighting. The details of the dataset are illustrated in Table 2.

### 4.2. Modeling

The proposed model is implemented using Python frameworks that were designed for machine learning and computer vision tasks. The main frameworks we use are TensorFlow, Keras, and Albumentations. The code was run on NVIDIA Tesla P100-PCIE-16 GB.

The bottom-up path of the model is similar to MobileNetV2. We use the technique of transfer learning to initialize the parameters of the bottom-up path of our model with the weight values of the original MobileNetV2 model that was trained on the ImageNet dataset [7]. The used version of the dataset is that used in ImageNet Large Scale Visual Recognition Challenge (ILSVRC) which contains 1000 classes of different objects. By the use of transfer learning, the model is faster to learn with a high learning quality, especially when we have a relatively small dataset.

The parameters of the top-down path, on the other hand, are initialized using the normal initialization [36]. To design the deep model, the choice and selection of the optimization and hyperparameters are given as follows: (i) training by using 150 epochs with a batch size of 10, (b) learning rate to be used is 5e-4, (c) loss function is a weighted combination between Dice loss and Focal loss, and (d) optimizer to be used is RMSProp.

The first row represents the street images. The second row contains the corresponding ground truth labels of the images in the first row. The ground truth labels are nothing but the predefined class labels of the captured image as per their pixel values. The classes represent the segment labels of the image.

### 4.3. Training and Testing Methods

In order to get the maximum performance of our model, we have to carefully choose training and testing conditions so that the parameters of the model are trained perfectly. The model is trained on random crops from a synthetic CamVid training dataset. Each image crop has dimensions of 320 × 320. After that, the model is tested on image frames of full size from the CamVid testing dataset. Three training issues we focus on in our research are as follows: data augmentation, parameter initialization methods, and choosing the optimization methods and training hyperparameters.

#### 4.3.1. Data Augmentation

Data augmentation is used to artificially expand the size of the available dataset by creating modified versions of the dataset items. If these items are image frames, like in our case, data augmentation techniques include many computer vision practices such as cropping, flipping, and so on. The main benefit of data augmentation is that it increases the diversity of the used dataset when obtaining new data is expensive in some way. Using data augmentation, the mathematical model can capture the data invariance during the training phase, and thus the resulting model has a higher ability to generalize so that it can correctly process new data. If data augmentation is used correctly, serious problems related to model training such as underfitting and overfitting can be eliminated.

As we mentioned earlier, CamVid dataset is better to be augmented so that our model can be trained better and can correctly do the semantic segmentation process on new images. To obtain high road scene diversity, the approach of data augmentation we use performs more excessive data augmentation than that used in the training process of the original UNet. The main data augmentation practices we use during the training phase include

- Horizontal flipping.
- Shift, scale, and rotate transformations.
- Random grid and optical distortions, random Gaussian noise, and random four-point perspective transform.
- Histogram equalization.
- Random brightness and contrast, random gamma transformation, random image sharpening, and random blur (normal, motion, median, and Gaussian blur). Hue saturation need to be deleted. Random image crop with dimension 320 x 320 is used for model training.

#### 4.3.2. Initialization

The bottom-up path of the model is similar to MobileNetV2. We use the technique of *transfer learning* to initialize the parameters of the bottom-up path of our model with the weight values of the original MobileNetV2 model that was trained on the ImageNet dataset [26]. The used version of the dataset is that used in ImageNet Large Scale Visual Recognition Challenge (ILSVRC) which contains 1000 classes of different objects. By the use of transfer learning, the model is faster to learn with a high learning quality, especially when we have a relatively small dataset.

The parameters of the top-down path, on the other hand, are initialized using He Normal initialization (https://keras.rstudio.com/reference/initializer_he_normal.html). In this method, the weights are initialized keeping in mind the size of the previous layer which helps in attaining a global minimum of the cost function faster and more efficiently. The algorithm of initialization using this method can be summarized as follows:

- First, initialize weights with values taken from a standard normal distribution.
- Second, multiply each weight value by $\sqrt{2}/\sqrt{n}$ where *n* is the number of incoming connections to the layer in which the parameter under initialization is located.
- Note that bias parameters are initialized to zero.

#### 4.3.3. Optimization and Hyperparameters

The basic design choices regarding optimization process and hyperparameters are as follows:

- We designed the training process to pass 150 epochs with a batch size of 10 frames (an epoch is one pass over the training dataset).
- The initial learning rate is *α*=5 *e* − 4.
- The used loss function is a weighted combination between 2 loss functions: Dice loss and focal loss.

The used optimizer is RMSProp optimizer.

## 5. Experimental Details

**Figure 5:**
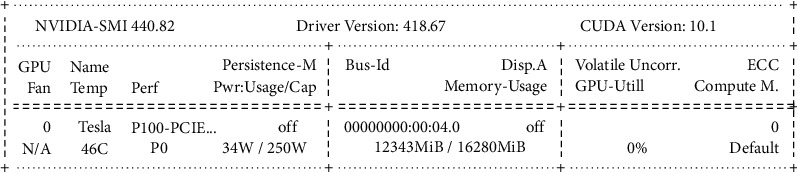
Details of hardware specifications used in the work.

The proposed model is implemented using Python frameworks that were designed for machine learning and computer vision tasks. The main libraries used are TensorFlow and Keras deep learning libraries including this Albumentationsis also being used in the work (https://albumentations.ai/). This is an efficient and user-friendly image augmentation Python library aimed at helping researchers to create fast augmentations based on a highly-optimized OpenCV library. The code was run on *NVIDIA Tesla P100-PCIE-16 GB*. The hardware specifications are presented in Figure 5. The training was done using CamVid dataset, where random image crops of 320 × 320 are used.

The model has been trained using the CamVid dataset followed by data augmentation. Random image crops of 320 × 320 were used to train the model. The model is trained for 150 epochs with an initial learning rate of *α* = 5*e* − 4 and a batch size of 10, and the RMSProp optimizer was used. The described model is tested on the CamVid test dataset and evaluated depending on a number of evaluation metrics. When choosing metrics for evaluation, we have to take into consideration the issue of class imbalance. In other words, although using the technique of class weighting during the training process can reduce the impact of class imbalance, the resulting model usually tends to perform better on classes of higher frequency. Therefore, we choose metrics so that they do some kind of averaging between results coming from the evaluation of each class separately. In addition, another metric that is related to measuring the model complexity is used.

The following metrics are used for the present work.

- Mean class accuracy (mCA): in semantic segmentation, the prediction accuracy can be defined as the number of correctly classified pixels over the total number of pixels. To ensure that class imbalance does not lead to misleading accuracy values, we follow the common practice of taking the average accuracy between all calculated accuracies of the defined classes. The resulting value is called mCA.
- Mean intersection over union (mIoU): this metric is common for computer vision classification tasks. In semantic segmentation tasks, intersection over union is defined as the number of pixel labels that are found in both the prediction frame and the ground truth frame over the pixel labels that are found in either the prediction frame or the ground truth frame. IoU is calculated for each segment class separately, and then the average between all classes is taken to be the mIoU.
- Number of parameters (#params): the complexity of deep learning models is very important if these models are intended to be used in real-time applications. The more complex a model is, the more the time needed for the model to calculate the output is. Therefore, the number of parameters used within a model is crucial to decide whether it is convenient to be implemented in a real-time application or not.

## 6. Results and Discussion

**Figure 6:**
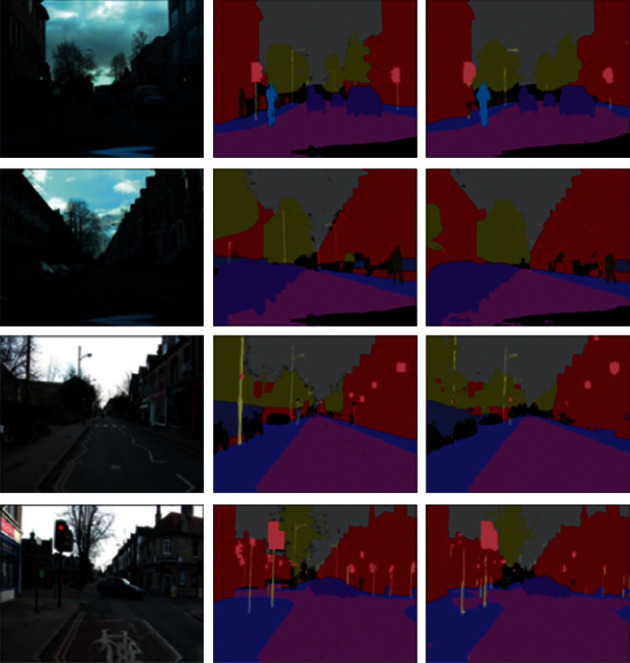
Samples of semantic segmentation results of the images tested on the CamVid dataset. The left column represents the original image, the column in the middle represents the ground truth labeled image, and the right column represents the predicted labels.

After training, the model uses the CamVid testing dataset as input and evaluates it using evaluation metrics. A mean class accuracy of 78.03% and mean intersection over union of 58.275% were obtained which makes our model a highly accurate semantic segmentation model especially with a relatively small number of parameters (5.2 M). The number of parameters used makes our model easy to be implemented as a part of a real-time autonomous system. Some samples of the semantic segmentation results of images tested on the CamVid dataset are shown in Figure 6. It represents some samples of semantic segmentation results of the images tested on the CamVid dataset used in the work. The left column represents the original image, the column in the middle represents the ground truth labeled image, and the right column represents the predicted labels.

We compare our designed model with other baseline models in terms of performance and complexity. The comparison results are shown in Table 3. As we notice, our proposed model provides a high performance with a relatively low number of parameters. The trade-off between the number of parameters and both mIoU and mCA is inevitable, and thus some models like DeepLab-LFOV and Dilated-8 would achieve a higher mIoU or mCA but are less applicable in real time due to the higher number of parameters. ENet and ESPNet have a lower number of parameters than ours but perform worse in terms of mIoU as models with a very small number of parameters may fail to capture the required complexity.

## 7. Conclusions

Semantic segmentation is a very important process for the perception of autonomous vehicles. It plays a major role in road scene understanding of the ego car. As deep learning methods have been improved during the last decade, more and more research is focusing on benefitting from deep learning so that better results can be obtained in all aspects of autonomous processes including perception and decision making. In this research work, we proposed a hybrid model that is built and trained upon the design principles of two deep learning models and used data augmentation techniques to increase the training quality. We aimed at making the designed architecture as accurate as real-time considerations could allow. The model was trained and tested on the CamVid dataset for which a high accuracy was obtained with a relatively small number of parameters. A comparison was done which proved the efficiency of our model compared with state-of-the-art models.

- In the future, more research can be conducted to increase the performance of the proposed hybrid design and overcome the current drawbacks. The main research directions that can be tested are as follows. The number of parameters of our model, despite being practical in real-time applications due to today's computing capabilities, can be made smaller by reducing the model complexity by taking into consideration the design principles of some other state-of-the-art models with a lower number of parameters.
- Better performance on the mIoU metric can be a topic of future research by considering some modern design approaches of CNNs and also the design choices of models that give a high mIoU.
- Other models different from MobileNetV2 can be made a backbone for the designed models. Some state-of-the-art architectures such as EfficientNet [16] represent a good candidate.

## Figures and Tables

**Table 1:** Details of bottleneck residual block.

Input	Operator	Output
*h* × *w* × *k*	1 × 1 conv2d, ReLU6	*h* × *w* × (*tk*)
*h* × *w* × *tk*	3 × 3 dwise, *s* = *s*, ReLU6	(*h*/*s*) × (*w*/*s*) × (*tk*)
(*h*/*s*) × (*w*/*s*) × *tk*	linear1 × 1 conv2d	(*h*/*s*) × (*w*/*s*) × *k*′

**Table 2:** Details of the dataset utilised for validating the proposed architecture.

Dataset	Videos with object class semantic labels (it presents ground truth labels of 32 semantic classes like building, tree, sky, side walk, column-pole, fence, pedestrian, and so on)
Name	CamVid
Size	604 MB

**Table 3:** Comparison between proposed model and other baseline models.

Model	mCA (%)	mIoU (%)	#Params (m)
SegNet-Basic [19]	62.9	46.2	—
SegNet [18]	65.2	55.6	29.5
FCN-8s [17]	—	57	134.5
ApesNet [24]	69.3	48	—
ENet [25]	68.3	51.3	0.36
ESPNet [26]	68.3	55.6	0.36
ESCNet [27]	70.9	56.1	0.185
DeepLab-LFOV [23]	—	61.6	37.3
Dilated-8 [22]	—	65.3	140.8
Proposed model	78.03	58.275	5.2

## Data Availability

The data used to support the findings of this study are available from the corresponding author upon request.
